# Smart Digital Solutions for EARLY Treatment of COGNitive Disability (EARLY-COGN^3): A Study Protocol

**DOI:** 10.3390/brainsci15030239

**Published:** 2025-02-24

**Authors:** Maria Cotelli, Francesca Baglio, Elena Gobbi, Elena Campana, Ilaria Pagnoni, Giovanna Cannarella, Alessandro Del Torto, Federica Rossetto, Angela Comanducci, Gennaro Tartarisco, Rocco Salvatore Calabrò, Simona Campisi, Raffaela Maione, Claudia Saraceno, Elisa Dognini, Sonia Bellini, Marta Bortoletto, Giuliano Binetti, Roberta Ghidoni, Rosa Manenti

**Affiliations:** 1 Neuropsychology Unit, IRCCS Istituto Centro San Giovanni di Dio Fatebenefratelli, Via Pilastroni, 4, 25125 Brescia, Italy; mcotelli@fatebenefratelli.eu (M.C.); ecampana@fatebenefratelli.eu (E.C.); ipagnoni@fatebenefratelli.eu (I.P.); gcannarella@fatebenefratelli.eu (G.C.); adeltorto@fatebenefratelli.eu (A.D.T.); rmanenti@fatebenefratelli.eu (R.M.); 2 IRCCS Fondazione Don Carlo Gnocchi—ONLUS, Via Alfonso Capecelatro, 66, 20148 Milan, Italy; fbaglio@dongnocchi.it (F.B.); frossetto@dongnocchi.it (F.R.); acomanducci@dongnocchi.it (A.C.); 3 National Research Council of Italy, Institute for Biomedical Research and Innovation, C/O via Leanza, Istituto Marino, Mortelle, 98164 Messina, Italy; gennaro.tartarisco@cnr.it (G.T.); simona.campisi@irib.cnr.it (S.C.); 4 IRCCS Centro Neurolesi “Bonino Pulejo”, Salita Villa Contino, 21, 98124 Messina, Italy; roccos.calabro@irccsme.it (R.S.C.); raffaela.maione15@gmail.com (R.M.); 5 Molecular Markers Laboratory, IRCCS Istituto Centro San Giovanni di Dio Fatebenefratelli, Via Pilastroni, 4, 25125 Brescia, Italy; csaraceno@fatebenefratelli.eu (C.S.); sbellini@fatebenefratelli.eu (S.B.); rghidoni@fatebenefratelli.eu (R.G.); 6 Neurophysiology Laboratory, IRCCS Istituto Centro San Giovanni di Dio Fatebenefratelli, Via Pilastroni, 4, 25125 Brescia, Italy; edognini@fatebenefratelli.eu (E.D.); marta.bortoletto@cognitiveneuroscience.it (M.B.); 7 Molecular Mind Lab, IMT School for Advanced Studies Lucca, Piazza San Francesco, 19, 55100 Lucca, Italy; 8 MAC-Memory Clinic and Molecular Markers Laboratory, IRCCS Istituto Centro San Giovanni di Dio Fatebenefratelli, Via Pilastroni, 4, 25125 Brescia, Italy; gbinetti@fatebenefratelli.eu

**Keywords:** digital health, cognitive training, non-pharmacological intervention, neurocognitive disorders

## Abstract

**Background:** Healthy cognitive functioning is a primary component of well-being, independence, and successful aging. Cognitive deficits can arise from various conditions, such as brain injury, mental illness, and neurological disorders. Rehabilitation is a highly specialized service limited to patients who have access to institutional settings. In response to this unmet need, telehealth solutions are ideal for triggering the migration of care from clinics to patients’ homes. **Objectives:** The aim of EARLY-COGN^3 will be threefold: (1) to test the efficacy of a digital health at-home intervention (tele@cognitive protocol) as compared to an unstructured cognitive at-home rehabilitation in a cohort of patients with Chronic Neurological Diseases (CNDs); (2) to investigate its effects on the biomolecular and neurophysiological marker hypothesizing that people with CNDs enrolled in this telerehabilitation program will develop changes in biological markers and cortical and subcortical patterns of connectivity; (3) to analyze potential cognitive, neurobiological, and neurophysiological predictors of response to the tele@cognitive treatment. **Method:** In this single-blind, randomized, and controlled pilot study, we will assess the short- and long-term efficacy of cognitive telerehabilitation protocol (tele@cognitive) as compared to an unstructured cognitive at-home rehabilitation (Active Control Group—ACG) in a cohort of 60 people with Mild Cognitive Impairment (MCI), Subjective Cognitive Complaints (SCCs), or Parkinson’s Disease (PD). All participants will undergo a clinical, functional, neurocognitive, and quality of life assessment at the baseline (T0), post-treatment (5 weeks, T1), and at the 3-month (T2) follow-up. Neurophysiological markers and biomolecular data will be collected at T0 and T1. **Conclusions:** EARLY-COGN^3 project could lead to a complete paradigm shift from the traditional therapeutic approach, forcing a reassessment on how CNDs could take advantage of a digital solution. (clinicaltrials.gov database, ID: NCT06657274)

## 1. Introduction

Dementia is currently a public health priority at the center of the global action plan (2017–2025), requiring new solutions to support patients and families in managing disabilities related to the disease [1]. The world’s population is aging, requiring us to face new challenges. Improvements in medical care have led to a consistent increase in the average age, and it is estimated that the elderly population will increase again. Healthy cognitive functioning is a primary component of well-being, independence, and successful aging. In this context, the importance of prompt intervention with rehabilitation on residual capabilities is well known. However, rehabilitation remains a highly specialized service for a limited number of subjects with access to an institutional setting [2]. In response to this unmet need, telehealth solutions are ideal for triggering the migration of care from clinics to individuals’ homes and, consequently, scaling up cognitive rehabilitation from a limited number of participants to a broader range of targets [3]. The need for alternative kinds of dementia service delivery is increasing because of the growing demand and cost of healthcare; the rapid development of technology could meet this need. In particular, equitable access to services, improvement of quality of care, and the promotion of self-management are benefits to be delivered from telerehabilitation.

The increase in life expectancy in recent decades has led to a large number of people living into old age, alongside an increased risk of developing Chronic Neurological Diseases (CNDs), such as neurodegenerative diseases [4,5,6]. A higher cumulative risk of dementia has been widely demonstrated in Mild Cognitive Impairment (MCI) and Subjective Cognitive Complaints (SCCs) subjects, and in Parkinson’s Disease (PD) patients, as compared to the general population [7,8,9]. As the disease progresses, individuals become increasingly dependent on medical services and family support. SCCs condition is defined based on self-reports regarding complaints of cognitive decline, often of memory, although normal functioning can be detected on objective tests [10,11]. The concept of SCCs is highly significant to the field of aging because this condition is a risk factor for developing dementia [11,12,13,14,15]. The diagnosis of MCI requires both subjective cognitive complaints and objective impairment in at least one area of cognition [16,17]. Given the increased risk of developing Alzheimer’s Disease (AD) in these populations [11], there is a strong argument for developing effective interventions aimed at reducing cognitive decline in MCI and SCCs. Regarding patients with PD, one of the most common age-related brain disorders, it is defined primarily as a movement disorder, with typical symptoms including resting tremor, rigidity, bradykinesia, and postural instability [18,19,20]. However, a range of non-motor symptoms emerge throughout the course of the disease, such as cognitive decline and mood disturbances [21]. In terms of cognitive deficits, a meta-analysis has reported that cognitive training could potentially help to attenuate cognitive deficits in patients with PD [22].

There have been recent arguments for a beneficial effect of non-invasive treatments such as cognitive programs in SCCs, MCI, and PD [23,24,25,26]. Given the limited effectiveness of pharmacological treatments, attention has been focused in recent years on non-pharmacological interventions aimed at preventing and treating cognitive deficits, as well as the associated difficulties with activities of daily living in individuals with neurodegenerative diseases. Among these, cognitive training offers a potential approach for dementia prevention and cognitive function improvement [27,28,29,30,31,32,33]. A critical aspect of cognitive training programs is that the most promising interventions have involved intensive in-person sessions that are unlikely to be cost-effective or feasible for large-scale implementation [34]. Within the framework of non-pharmacological interventions, the use of technology to assist at-home people at risk of or with mild dementia has gradually gained importance [3,35]. Telerehabilitation technologies enable the provision of health services remotely, allowing participants living in rural areas or those with mobility difficulties access to healthcare [3,35,36,37,38,39,40,41,42,43,44,45,46,47,48,49,50,51,52,53,54]. Moreover, telerehabilitation offers the advantage of providing rehabilitation within the subject’s natural home environment, making the treatment more realistic and potentially more generalizable to the individual’s daily life [55].

Cognitive rehabilitation is a potential approach for improving cognitive function and delaying cognitive decline [22,27,28,29,56,57,58,59,60,61,62,63,64,65,66,67,68]. In the case of neurodegenerative disease, what makes the situation more challenging is that subjects are usually diagnosed and treated only after symptoms appear, typically 10–20 years after the initial pathological changes in the brain. This delay makes it difficult to counteract disease progression. Therefore, early rehabilitation has a crucial role in managing neurodegenerative conditions and it is extremely important to identify effective interventions capable of reducing the incidence of these disabilities in older adults with CNDs [69,70]. The potential benefits of telerehabilitation have been highlighted in the literature [71] and these technologies have been also applied to cognitive rehabilitation in individuals with neurodegenerative diseases or with subjective complaints [3,40,51,54,72,73], with the aim to prevent or delay the onset of CNDs [74]. The delivery of rehabilitation through various technologies can help overcome the limitations of high-intensity face-to-face (FTF) rehabilitation interventions [3,35,36,40,41,75]. The results of studies showing that telerehabilitation achieves comparable outcomes to traditional FTF services appear interesting [42,43,44,45,46,47,48,49,51,52,53,54,76,77,78,79], and two systematic reviews [38,51] reported that cognitive telerehabilitation has similar effects in terms of efficacy, validity, and reliability to that of conventional FTF rehabilitation. In a recent study, we observed that MCI subjects who had received FTF rehabilitation, followed by asynchronous tablet telerehabilitation, maintained improvements in memory at the 7-month follow-up [80]. This Randomized Clinical Trial (RCT) confirms the feasibility, patient adherence to treatment, and good usability of technology in subjects with MCI. These results are in agreement with recent findings and meta-analyses on the efficacy and feasibility of a cognitive telerehabilitation program in individuals with MCI [46,48,76,78,81,82,83,84]. Regarding PD, telemedicine could represent an emerging and innovative approach to support cognitive and behavioral rehabilitation, reducing the overload of healthcare facilities and promoting home-based therapy [40,77,85,86,87]. Additionally, the implementation of digital contents promotes engagement [88] and facilitates measurement of the patient’s performance and progress in terms of accuracy, reaction time, number of repetitions, and time spent in rehabilitative sessions.

Nevertheless, asynchronous models of telerehabilitation require a complex technological ecosystem underlying clinic–home communication, and, as a result, RCTs demonstrating the effectiveness of these solutions are still scarce [72,89]. However, no studies have been conducted to assess the feasibility and efficacy of cognitive rehabilitation delivered by digital therapeutics—an additional way to scale up cognitive rehabilitation—in older people with CNDs.

The neural bases of spontaneous and rehabilitation-induced recovery remain largely unknown. While there is substantial evidence that brain plasticity plays a role in overcoming cognitive deficits, until recently rehabilitation had been based only upon observational studies [90]. Within the emerging field of cognitive neuroscience, recent discoveries suggest that the central nervous system responds dynamically to insults, thus explaining that, at least in some cases, lost behaviors can be restored [91].

Many studies have investigated various cognitive, neurobiological, and neurophysiological predictors of response to rehabilitation intervention. However, a synthesis of this literature and an understanding of which cognitive, biological, and neurophysiological targets predict response is lacking. Neurophysiological response to Transcranial Magnetic Stimulation (TMS) and blood biomarkers of neurodegeneration, neuroinflammation, synaptic function, and neuronal communication might influence the efficacy of cognitive rehabilitative protocols based on a digital health at-home intervention [92].

In this single-blind, randomized, controlled pilot study, we will aim to: (1) test the short-term and long-term efficacy of a cognitive telerehabilitation protocol (tele@cognitive) as compared to an unstructured cognitive at-home rehabilitation (Active Control Group—ACG) in the treatment of a cohort of patients with SCCs, MCI, PD; (2) investigate its effects on the biomolecular and neurophysiological markers hypothesizing that people with CNDs enrolled in this telerehabilitation program will develop changes in biological markers and in cortical and subcortical patterns of connectivity; (3) analyze potential cognitive, neurobiological, and neurophysiological predictors of response to the tele@cognitive treatment.

In particular, the first aim will be to assess the short-term and long-term efficacy of tele@cognitive protocol as compared to an unstructured cognitive at-home rehabilitation in the treatment of a cohort of patients with CNDs, monitoring intervention effectiveness by a clinical, functional, neurocognitive, and quality of life assessment applied before treatment (T0), after treatment (5 weeks, T1), and at a 3-month follow up visit (T2). We hypothesize that this digital health at-home intervention would ameliorate cognitive abilities more than unstructured at-home rehabilitation in subjects with SCCs, MCI, and PD. We will also evaluate adherence to the rehabilitation program delivered at-home in the absence of therapist supervision. It is hypothesized that, at an early stage of the disease, cognitive telerehabilitation treatment with innovative digital solutions can bring about positive effects on treatment adherence and difficulties specific to each clinical condition.

The second aim will be to investigate the effects of a cognitive rehabilitative protocol based on a digital health at-home intervention (tele@cognitive) on biomolecular and neurophysiological markers, hypothesizing that people with CNDs enrolled in the tele@cognitive protocol will develop changes in circulating biomarkers and patterns of connectivity. Specifically, at T0 and T1, we will study the effect of tele@cognitive treatment on: i) blood biomarkers reflecting neurodegeneration, amyloid-β, tau and alpha-synuclein pathologies, neuroinflammation, synaptic function, and neuronal communication; ii) neurophysiological responses to TMS over the motor cortex. This approach will give a broad overview of the circuits modulated through telerehabilitation (tele@cognitive treatment), possibly highlighting differential effects between the groups.

Finally, the third aim will be to investigate potential cognitive, neurobiological, and neurophysiological predictors of treatment outcomes in people with CNDs who received innovative technology-enhanced telerehabilitation intervention (tele@cognitive group).

## 2. Materials and Methods

**Figure 1:**
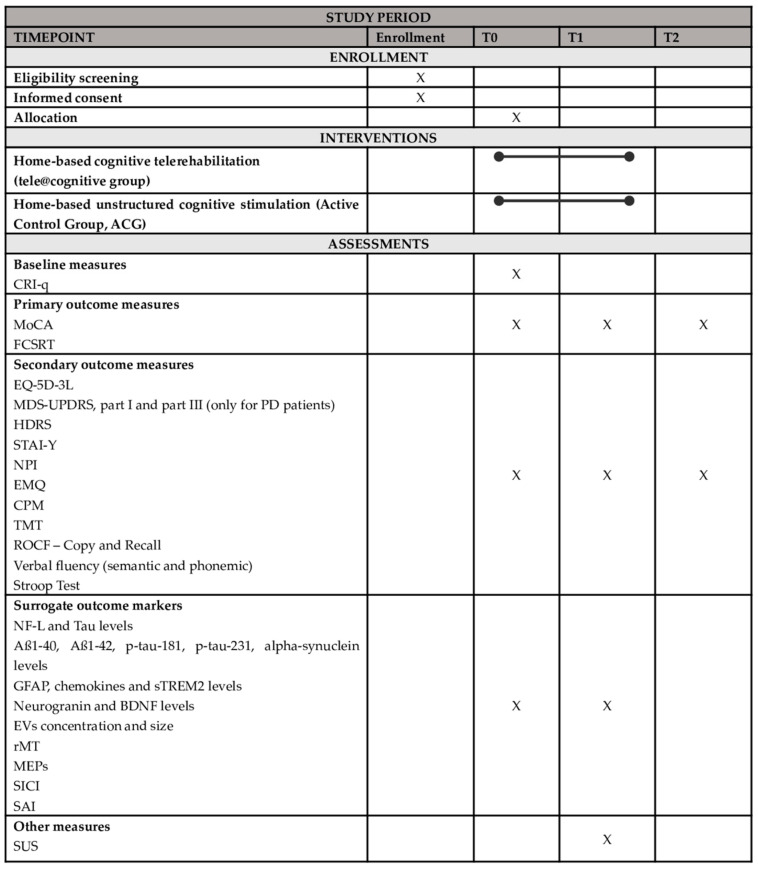
SPIRIT figure for the enrollment, intervention, and assessment schedule in the study. Aß = Amyloid β; BDNF = Brain-Derived Neurotrophic Factor; CPM = Raven’s Colored Progressive Matrices; CRI-q = Cognitive Reserve Index—questionnaire; EMQ = Everyday Memory Questionnaire; EQ-5D-3L = 3-level EQ-5D version; EVs = Extracellular Vesicles; FCSRT = Free and Cued Selective Reminding Test; GFAP = Glial Fibrillary Acidic Protein; HDRS = Hamilton Depression Rating Scale; MDS-UPDRS = Unified Parkinson’s Disease Rating Scale; MEPs = Motor Evoked Potentials; MoCA = Montreal Cognitive Assessment; NF-L = Neurofilament light chain; NPI = Neuropsychiatric Inventory; PD = Parkinson’s Disease; rMT = resting Motor Threshold; ROCF = Rey–Osterrieth Complex Figure; SAI = Short-latency afferent inhibition; SICI = Short-interval intracortical inhibition; STAI-Y = State-Trait Anxiety Inventory; sTREM2 = soluble Triggering receptor expressed on myeloid cells 2; SUS = System Usability Scale; T0 = Baseline Assessment; T1 = Post-treatment Assessment; T2 = 3-month follow-up Assessment; TMT = Trial Making Test.

The protocol of the present study has been developed, as outlined in the “Standard Protocol Items: Recommendations for Interventional Trials” (SPIRIT) guidelines [93] (Figure 1).

The study will be conducted in accordance with the Declaration of Helsinki. It has been approved by the Ethics Committee (Comitato Etico territoriale Lombardia 6, Italy; protocol number: 037883/24) of the Principal Investigator (IRCCS Istituto Centro San Giovanni di Dio Fatebenefratelli, Brescia). All patients must give their written informed consent before participating in the study. This study was registered in the clinicaltrials.gov database (identifier: NCT06657274) on 23 October 2024.

### 2.1. Trial Design

This multicentric study is a single-blinded, randomized, controlled pilot study (RCT) involving 60 subjects with CNDs (MCI, SCCs, and PD) recruited from IRCCS Istituto Centro San Giovanni di Dio, Fatebenefratelli, Brescia, IRCCS Fondazione Don Carlo Gnocchi—ONLUS, Milan, Italy and IRCCS Centro Neurolesi “Bonino Pulejo”, Messina, Italy.

**Figure 2:**
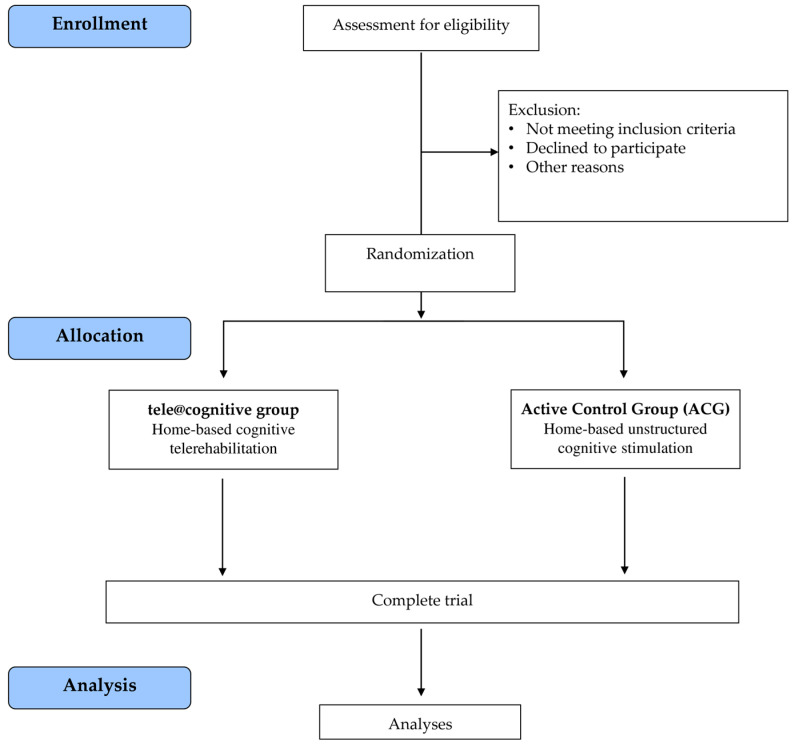
Trial work plan.

After recruitment, the subjects will be randomized into one of two parallel groups: (i) the tele@cognitive group, which will receive home-based cognitive telerehabilitation (tele@cognitive treatment); (ii) the Active Control Group (ACG), which will receive home-based unstructured cognitive stimulation. All participants will undergo a clinical, functional, neurocognitive, and quality of life assessment at the baseline (T0), post-treatment (5 weeks, T1), and at the 3-month (T2) follow-up. Neurophysiological markers (TMS) and biomolecular data will be collected at T0 and T1. The trial work plan is shown in Figure 2.

### 2.2. Randomization and Blinding

The assigned group will be obtained by stratified randomization (1:1 ratio), according to clinical condition, age, and his/her performance in the Montreal Cognitive Assessment (MoCA) test [94,95,96]. Stratified randomization will be achieved by generating a separate block for each combination of covariates, and participants will be assigned to the appropriate block of covariates by a researcher blinded to the study’s aims. Details of the allocated group will be given on cards contained in sequentially numbered, opaque, and sealed envelopes. A blinded researcher will conduct the evaluations.

### 2.3. Sample Size

In the RCT, patients will be assigned to either receive tele@cognitive protocol or a home-based unstructured cognitive stimulation (ACG). The sample size of the present study was estimated on the basis of a previous work on people within the Alzheimer’s Disease continuum [3] and it was computed on a primary outcome variable (an increase in MoCA score). Applying a two-tailed independent samples *t*-test (alpha = 0.05, beta = 0.80) and considering the effect size (d = 0.784) between two types of intervention (tele@cognitive vs. ACG), we obtained a minimum sample size of 60 subjects (30 per group), taking into account the drop-out rate.

### 2.4. Participants

According to the sample size calculation, the EARLY-COGN^3 trial has a target enrollment of 60 subjects with CNDs (MCI, SCCs, and PD).

#### Inclusion and Exclusion Criteria

Formal criteria-based diagnosis of MCI, SCCs, and PD will be applied.

Patients will include both male and female subjects affected by CNDs: MCI, SCCs and PD.

Inclusion criteria will include: (1) age eligible for the study: 18 ≤ age ≤ 85; (2) agreement to participate by signing the informed consent form; (3) diagnosis of PD (Hoehn & Yahr < 3 [97]), MCI (with Cognitive Dementia Rating Scale - CDR ≤ 0.5 [98] and Mini Mental State Examination - MMSE raw score ≥ 24 [99,100,101]), or SCCs; (4) native Italian speakers; (5) MoCA-corrected score ≥ 17.36 [95]; (6) education ≥ 5 years; (7) availability of a caregiver/study partner able to support the participant; (8) absence of marked hearing/visual impairment; (9) no rehabilitation program in place at the time of enrollment or in the last 3 months before enrollment; (10) stable drug treatment (last 3 months) with L-Dopa or dopamine agonists (PD group) and acetylcholinesterase (MCI group), if any.

Key exclusion criteria will include: (1) the presence of any medical or psychiatric illness that could interfere with completing assessments; (2) the presence of any medical or psychiatric illness that could make the administration of TMS unsafe.

### 2.5. Intervention Procedures

The cognitive rehabilitation protocol (5 weeks, 3 sessions/week, 45 min/session) will be delivered to all participants according to the corresponding experimental group:

- 30 CNDs patients will receive home-based cognitive rehabilitation activities with an innovative digital solution for remote rehabilitation of cognitive difficulties (tele@cognitive group);
- 30 CNDs patients will receive home-based unstructured cognitive rehabilitation treatment (Active Control Group—ACG).

The technological solution that will be applied in the tele@cognitive group consists of: (a) a telemedicine platform (RICORDO platform), which allows prescription and planning of digital content for cognitive rehabilitation and monitoring of the rehabilitation cycle and (b) RICORDO application [http://www.ricordo-dtx.com/ (accessed on 10 January 2025)] that will be downloaded on tablets provided to patients by the recruiting units, which will allow the patient to benefit from personalized rehabilitation treatment remotely and in asynchronous mode. In detail, through RICORDO platform, the clinician will have the possibility of prescribing, within the patient’s Individual Rehabilitation Plan (PRI), a personalized intervention that will involve the use of libraries of digital rehabilitation content that maps all the neurocognitive domains provided for in the most recent version of the Diagnostic and Statistical Manual of Mental Disorders (DSM-5) [102]. In particular, the rehabilitation program will include cognitive activities focused on cognitive domains, such as executive functions, memory (long-term memory of both verbal and visuospatial type, autobiographical memory, and semantic memory), attention (selective attention and divided attention), and spatial–temporal orientation and language. Each cognitive exercise will be structured into five levels of difficulty that adaptively increase (algorithms on both subject’s performance and perceived difficulty). All data on patient progress and adherence will be collected and will be available to evaluate and monitor the results of rehabilitation.

The unstructured cognitive rehabilitation treatment applied in the ACG will offer paper–pencil cognitive activities that will stimulate the same cognitive abilities indicated in the innovative digital solution. Subjects assigned to ACG will be requested to work for the same duration and frequency as the tele@cognitive group; they will receive from the therapist an instructions booklet and a participant diary.

### 2.6. Outcome Measures

#### 2.6.1. Primary Outcome Measures

The primary outcome measures for this study will be the change from the baseline (T0) to post-treatment (T1) and 3-month follow-up visit (T2) in:

1. Montreal Cognitive Assessment (MoCA). MoCA [96] is a screening test for global cognitive functioning. It includes tasks involving several cognitive domains: visuospatial, executive function, naming, selective and sustained attention, language, abstraction, memory, and orientation (score range min = 0, max = 30, higher score = better outcome).
2. Long-term episodic verbal memory as assessed by Free and Cued Selective Reminding Test (FCSRT). FCSRT [103] is a measure of long-term episodic verbal memory. It provides five scores: Immediate Free Recall (IFR, spontaneous recall across three trials; score range min = 0, max = 36), Immediate Total Recall (ITR, total recall across three trials; score range min = 0, max = 36), Delayed Free Recall (DFR, score range min = 0, max = 12), Delayed Total Recall (DTR, score range min = 0, max = 12). Higher scores indicate better performance. Finally, the Index of Sensitivity to Cueing (ISC, score range min = 0, max = 1) reflects the difference between the number of items recalled spontaneously and the number of items recalled with the help of cues. A higher ISC indicates a greater sensitivity to cues.

#### 2.6.2. Secondary Outcome Measures

The secondary outcome measures for this study will be the change from the baseline (T0) to post-treatment (T1) and 3-month follow-up visit (T2) in:

1. Quality of life as assessed by EQ-5D-3L. EQ-5D-3L [104] is a measure of health status consisting of five dimensions (mobility, self-care, usual activities, pain/discomfort, and anxiety/depression). Each dimension has three response levels (no problems, some problems, unable to/extreme problems). In addition, the questionnaire includes a visual analog scale that records the patient’s perception of his or her overall health (score range min = 0, max = 100, higher score = better outcome).
2. Non-motor experiences of daily living as assessed by MDS-UPDRS scale, part I. MDS-UPDRS scale [105], part I (only for the PD group) is a measure of non-motor experiences of daily living consisting of a total of 13 questions (score range min = 0, max = 52, higher score = worse outcome).
3. Motor abilities as assessed by MDS-UPDRS scale, part III. MDS-UPDRS scale [105], part III (only for the PD group) is a measure of motor abilities consisting of a total of 18 questions with 33 individual items. Each item has a 0–4 rating, where 0 = normal, 1 = slight, 2 = mild, 3 = moderate, and 4 = severe (score range min = 0, max = 132, higher score = worse outcome).
4. Depressive symptoms as assessed by Hamilton Depression Rating Scale (HDRS). HDRS [106] is a measure of severity of the depressive symptoms consisting of a total of 21 items (score range min = 0, max = 64, higher score = worse outcome).
5. Anxiety symptoms as assessed by State-Trait Anxiety Inventory (STAI-Y). The STAI-Y [107] consists of two 20-item scales providing separate measures of state and trait anxiety (S-Anxiety and T-Anxiety). Each scale has a score range from a minimum of 20 to a maximum of 80, with a higher score on the scale indicating a worse outcome.
6. Behavior and personality as assessed by Neuropsychiatric Inventory (NPI). NPI [108] is used for assessing various neuropsychiatric symptoms. The inventory consists of 12 core domains, each reflecting specific neuropsychiatric symptoms. Each symptom is rated for both frequency and severity (score range min = 0, max = 144, higher score = worse outcome).
7. Memory complaints as assessed by Everyday Memory Questionnaire (EMQ). EMQ [109] is a 20-item questionnaire that evaluate the frequency and impact of memory problems in daily life (score range min = 20, max = 180, higher score = worse outcome).
8. Non-verbal abstract reasoning as assessed by Raven’s Colored Progressive Matrices (CPM). CPM [110] is a measure of non-verbal abstract reasoning (score range min = 0, max = 36, higher score = better outcome).
9. Attentional abilities as assessed by Trial Making Test (TMT). TMT (Part-A and Part-B) [111] is a measure of attentional abilities, visuo-conceptual and visual–motor tracking. TMT-Part A involves visual scanning, number recognition, number sequencing, and motor speed. TMT-Part B assesses mental flexibility in managing more than one stimulus at a time and in shifting the course of an ongoing activity. High execution times indicate poor performance (score range min = n/a, max = no limits).
10. Executive abilities as assessed by Stroop Test. Stroop Test [112] is a measure of executive abilities, including visual attention, selective attention, cognitive flexibility, and inhibitory control of behavior. Two scores are calculated with consideration of the number of errors and the time taken to complete all parts. High execution times and high numbers of errors indicate poor performance.
11. Constructional praxia as assessed by Rey–Osterrieth Complex Figure-Copy (ROCF). ROCF [113] is a measure of constructional praxia (score range min = 0, max = 36, higher score = better outcome).
12. Fluency abilities as assessed by Verbal Fluency (semantic and phonemic). Verbal Fluency (semantic and phonemic) [114] is a measure of verbal and semantic fluency abilities, executive functions abilities, lexical store size, lexicon access, and lexical organization (score range min = 0, max = no limits, higher score = better outcome).
13. Nonverbal long-term memory as assessed by ROCF-Recall. ROCF-Recall [113] is a measure of nonverbal long-term memory (score range min = 0, max = 36, higher score = better outcome).

#### 2.6.3. Surrogate Outcome Measures

The surrogate outcome measures will be the change from the baseline (T0) to post-treatment (T1) in:

1. Neurofilament light chain (NF-L) and Tau levels.
2. Aß1-40, Aß1-42, p-tau-181, p-tau-231, and alpha-synuclein levels.
3. Glial Fibrillary Acidic Protein (GFAP), chemokines, and sTREM2 levels.
4. Neurogranin and Brain-Derived Neurotrophic Factor (BDNF) levels.
5. Concentration and size of plasma Extracellular Vescicles (EVs).
6. Resting Motor Threshold (rMT). rMT reflects membrane excitability of corticospinal neurons. Lower values indicate increased excitability.
7. Motor Evoked Potentials (MEPs). MEPs are muscle twitches resulting from complex descending corticospinal volleys occurring after TMS pulses. MEP amplitude and latency reflect the integrity of the corticospinal tract and corticospinal excitability.
8. Short-interval intracortical inhibition (SICI). TMS-SICI reflects inhibitory interneuronal circuits acting via GABA A receptors [115].
9. Short-latency afferent inhibition (SAI). TMS-SAI reflects cholinergic transmission [116].

### 2.7. Data Collection

Demographic characteristics and cognitive reserve, evaluated with the Cognitive Reserve Index—questionnaire (CRI-q [116]), will be collected at the baseline evaluation (T0). The same trained neuropsychologist (blinded to patient treatment allocations) will assess primary and secondary outcome measures for a single patient in all visits (T0, T1, and T2). Neurophysiological and biomolecular data will be collected only at the baseline (T0) and at post-treatment (at 5 weeks, T1). A subjective assessment of the digital system’s usability will be performed at T1 using the System Usability Scale (SUS) [117], a 10-item attitude Likert scale. Best practices and standards will be adopted for the collection, storage, and treatment of the data. Clinical, functional, neurocognitive, and quality of life assessment data will be collected by clinicians and stored in a specific dataset. Data will be pseudo-anonymized so that they will not be linkable to the identities of the subjects. Neurophysiological data will be organized according to Brain Imaging Data Structure (BIDS) for non-invasive brain stimulation studies and safely stored in a dataset. Biological samples collected at T0 and T1 will be stored at −80 °C until the analyses. Biomolecular data will be safely stored in a dataset. Informed consent will be obtained from patients for the storage of all data. In more detail, the data collection in terms of data collected and tools will include:

[AIM1] Clinical, functional, neurocognitive, and quality of life data recorded at T0 (baseline), T1 (post-treatment, at 5 weeks), and T2 (3-month follow-up visit).

[AIM2] Neurophysiological data and blood samples will be collected at T0 and T1. Specifically:

- Aß1-40, Aß1-42, p-tau-181, p-tau-231, NF-L, tau, GFAP, BDNF, Eotaxin-1, Eotaxin-3, alpha-synuclein, neurogranin, and sTREM2 will be measured in plasma or serum by commercially available kits. Coefficients of Variation (CVs) within-run will be accepted when in agreement with information from kit vendors. The concentration and size distribution of EVs will be assessed in light scattering mode with NanoSight NS300 instrument (Malvern, Worcestershire, United Kingdom). Samples will be diluted to obtain an optimal range of 20–150 particles/frame. For each sample, 5 videos of 60″ will be recorded and data will be processed using NanoSight NTA 3.2 software. Optimized post-acquisition settings will be kept constant during the analysis of all samples. Data obtained will be analyzed by comparing concentration (particles/mL), size distribution (nm), and their ratio (conc./size).
- In each TMS recording session, i.e., at T0 and at T1, CNDs participants will be comfortably seated in a dimly lit room and neurophysiological measures will be collected while they are at rest: (a) the resting motor threshold (rMT), defined as the lowest transcranial stimulus intensity at which TMS of motor cortex produces an EMG response in half of the trials, will be estimated with a probability density function; (b) motor-evoked potentials (MEPs) will be collected at stimulation intensity equal to 120% of the resting motor threshold; (c) SICI will be obtained by delivering a TMS pulse at 70% of rMT at 1, 2, 3, 5 ms before the test stimulus; (d) SAI will be measured as change in MEPs when TMS is delivered after the electrical stimulation of the median nerve at different intervals (−28, −24, −20, −16 ms). These measures will be collected by targeting the cortical motor hotspot of the first dorsal interosseous muscle (FDI) on the left hemisphere while measuring responses from muscles of the hand. The position of the coil will be monitored throughout the duration of the experiment, thanks to a neuronavigation system that allows the coil to remain in correspondence with the target area.

[AIM3] By employing a diverse range of data collection instruments and analysis techniques, we aimed to gain a comprehensive understanding of treatment effects on cognitive function. In particular, this multifaceted approach involves capturing and analyzing various aspects related to cognitive performance: clinical, functional, neurocognitive, and quality of life measures, biochemical data derived from blood samples, different parameters derived from TMS experiments, and the data gathered during the rehabilitation program.

### 2.8. Statistical Analysis

A repeated-measures analysis will be used to compare pre-treatment (T0) versus post-treatment (T1) and follow-up (T2) clinical, functional, neurocognitive, and quality of life variables for the two groups. Neurophysiological and biochemical data will be analyzed using repeated-measures ANOVAs by comparing the data recorded at T0 and T1. Statistical tests suitable for the data type will be employed to assess the differences between the groups. For continuous variables, independent *t*-tests will be utilized, while chi-square tests will be applied for categorical variables [117,118]. This analysis will verify the effectiveness of the randomization procedure, ensuring that any subsequent comparisons or evaluations between the groups are reliable and valid.

Within-group analyses will be conducted for the tele@cognitive and ACG groups to examine changes from T0 to T1 and T2. Paired *t*-tests or Wilcoxon signed-rank tests, depending on data distribution, will be employed to assess the effectiveness of the intervention over time by analyzing the variations in the collected multimodal parameters [118,119]. Furthermore, a between-group analysis will be conducted to quantitatively compare the outcomes of the two groups. Statistical tests such as independent *t*-tests, analysis of covariance (ANCOVA), or non-parametric tests will be employed, depending on the distributional assumptions and nature of the outcome variables [118,120].

In addition to these statistical analysis, predictive modeling will be implemented to identify key factors associated with treatment outcomes by integrating multimodal data from cognitive assessment, neurophysiological evaluations, biomolecular patterns, and user interaction data within a sophisticated AI framework. Given the heterogeneous nature of the data collected from multiple sources, feature selection is essential to identify the most informative features and reduce dimensionality. This step is critical to eliminate redundant or irrelevant variables and improve model performance [121]. Statistical methods such as chi-square tests and mutual information will be employed to identify significant predictors associated with positive treatment outcomes [122]. Specifically, the dataset will include baseline clinical, functional, neurocognitive, and quality of life measures, biomolecular markers, and neurophysiological parameters.

Data will be preprocessed to clean and normalize them appropriately. These will include handling missing values (e.g., using imputation for continuous variables and mode replacement for categorical variables), standardizing features to a common scale, and encoding categorical variables as needed. Machine learning algorithms, such as random forests, support vector machines, naive bayes classifiers, and ensemble techniques, will be applied to develop predictive models for treatment outcomes using the collected information [123]. Model performance will be assessed using cross-validation techniques like leave-one-out or Kfold, and parameters will be fine-tuned using grid search techniques to optimize model performance [124]. To ensure generalizability, performance will also be evaluated using metrics such as accuracy, precision, recall, and area under the ROC curve (AUC-ROC), depending on the nature of the prediction task.

For tasks involving high-dimensional or complex data, such as neurophysiological signals or time-series patterns, deep learning architectures (e.g., convolutional neural networks for spatial data, recurrent neural networks for temporal data) will be employed. These models will be customized and optimized using grid search to ensure they are well-suited to the specific characteristics of the data.

After model training, explainable AI (XAI) techniques, such as SHAP (Shapley Additive exPlanations) values will be employed to interpret the model’s predictions and quantify the contribution of each feature to the outcome [125]. SHAP values provide a unified measure of feature importance by assessing the impact of individual features on the model’s output, not only enhancing the clinical interpretability and transparency of the predictive models but also providing insights into which factors are most influential in driving treatment outcomes.

## 3. Discussion

Over the past years, several cognitive interventions, aiming to delay cognitive decline in older people and in people who are at risk of neurodegenerative disease or in the early stages of the disease, have been developed [27,126,127,128,129,130,131,132,133,134,135].

Several areas of research provide evidence that support the utility of cognitive interventions—both for prevention [53,73,136,137] and to provide therapeutic benefits to individuals in the early stages of neurocognitive disorders—referring to the concept of neuronal plasticity [138,139,140,141]. Neural plasticity is a lifelong process and several studies have shown that learning new associations, the exposure to novel stimuli, and enriched environments can induce synaptogenesis, synaptic remodeling, and neurogenesis in the adult rodent brain [142,143,144,145]. Accordingly, a complex relationship exists between neurodegenerative pathology and the genetic, epigenetic, functional, metabolic, and brain structural modifications that have affected the human brain during evolution. Individuals with greater cognitive reserve have been described as characterized by a reduced risk of developing dementia compared to individuals thought to have less cognitive reserve [146,147,148], and better cognitive performance [148,149,150,151]. Since cognitive reserve appears to have such a strong effect on the risk of developing dementia, cognitive rehabilitation, which targets cognitive reserve and compensatory reorganization mechanisms, may be clinically effective [152,153,154] and could attenuate cognitive decline in individuals at risk of developing dementia and potentially delay the onset of dementia and, at least in part, ameliorate the course of cognitive decline related to neurodegeneration once the diagnosis of dementia has been made [138].

An increasing need for alternative health service delivery is driven by the growing cost and demand for healthcare and the rapid development of technology.

Based on these considerations, the aim of EARLY-COGN^3 will be threefold: (1) to test the short-term and long-term efficacy of the tele@cognitive protocol as compared to an unstructured cognitive at-home rehabilitation in the treatment of a cohort of patients with CNDs; (2) to investigate its effects on the biomolecular and neurophysiological marker hypothesizing that people with CNDs enrolled in this telerehabilitation program will develop changes in biological markers and cortical and subcortical patterns of connectivity; (3) to study potential cognitive, neurobiological, and neurophysiological predictors of response to the tele@cognitive treatment.

Telerehabilitation refers to the use of Information and Communication Technologies (ICT) [35,36] and represents an innovative approach to overcome the obstacles associated with face-to-face intervention. Telerehabilitation technologies allow remote provision of services in patients’ homes or other environments, allowing access to healthcare to patients living in rural settings or with mobility difficulties [37,42,45,155,156,157,158]. In addition, the telerehabilitation modality offers the advantage of providing rehabilitation within the natural environment of the patient’s home, making the treatment more realistic and possibly more generalizable to the person’s daily life [55]. Equitable access to services, improved quality of care, ongoing intervention, and the promotion of self-management are several benefits to be delivered from telerehabilitation. A variety of technologies are now available to support the continuum of care for people with cognitive impairment or those who want to prevent it [3,51,53,72,73,80,85,136,137,159,160,161,162,163,164]. A systematic review showed a tendency for these services to lead to clinical improvements that are at least equal to those resulting from conventional rehabilitation programs [44].

The implementation of digital contents promotes engagement [88] and facilitates measurement of the patient’s performance and progress in terms of accuracy, reaction time, number of repetitions, and time spent in rehabilitative sessions. Asynchronous models of telerehabilitation require a complex technological ecosystem underlying clinic-home communication and, consequently, RCTs demonstrating the effectiveness of these solutions are still scarce [72,89].

As described, the RICORDO digital therapeutic aims to enhance several cognitive functions, including executive functions and attention. Executive functions include higher cognitive control functions such as working memory, inhibition, and cognitive flexibility skills that play an essential role in maintaining autonomy on a day-to-day basis and are essential prerequisites to new learning. Innovative studies exploring the possible impact of executive function enhancement using artificial intelligence chatbots appear to be very interesting. Future research is needed to test the effectiveness of these technologies in different clinical settings [165,166,167].

We expect that this RCT study will pave the way for our telerehabilitation protocol to be regarded as an innovative approach to scale up healthcare services for the broad population of individuals with CNDs, answering the needs of both patients and the healthcare system. It is well known that a critical point is the need for prompt interventions in response to neurodegeneration in people with CNDs. We expect that an early intervention will promote functional retention in daily activities while counteracting neurodegeneration. Moreover, one of the main challenges related to the rehabilitation of people with CNDs is the short-term nature of its effects.

In this context, our telerehabilitation protocol represents an ideal solution to provide intensive, long-lasting cognitive rehabilitation and to sustain treatment adherence, as well as to modify daily care routines. We expect that the findings of the present RCT will result in a recommendation of the use of a usable, safe, and effective way for changing CNDs’ behavior patterns, triggering mechanisms at the neurophysiological and neurobiological levels. The added value of this trial consists of: (i) adopting an asynchronous model of care out of the clinic, bypassing typical barriers obstructing accessibility to care structures and lowering system overburdening and costs; (ii) investigating the potential neurobiological and neurophysiological markers of the effects of the at-home tele@cognitive treatment; (iii) investigating potential cognitive, neurobiological, and neurophysiological predictors of response to the treatment, helping in the development of useful treatment pathways for patients with CNDs. It is necessary to clarify which types of patients can benefit the most from rehabilitation interventions and, above all, to pursue a more precise characterization of the specific intervention procedures. However, we acknowledge that the present RCT study has some limitations. In particular: a larger sample size might allow us to account for individual differences that could influence the efficacy of the treatment; we have not planned a cost-effective analysis of the telerehabilitation approach [168]; longer follow-up visits should be useful in following the progress of the improvement obtained after treatment. Moreover, it would have been useful to administer a scale that measured subjective experience and the attractiveness (hedonic quality) of the technology (e.g., UEQ scale; [169,170]).

## 4. Conclusions

At present, pharmacological therapies to prevent cognitive decline have not had great success and, consequently, much emphasis has been placed on non-pharmacological cognitive intervention to stimulate cognitive abilities and prevent functional decline.

EARLY-COGN^3 project could lead to a complete paradigm shift from the traditional therapeutic approach, forcing a reassessment on how CNDs could take advantage of a digital solution. Being software-based, our telerehabilitation protocol will allow clinicians to remotely collect data on patient progress and adherence, allowing a better customization of the rehabilitation program. Our telerehabilitation protocol would give the opportunity to target the need for rehabilitation of CNDs, which is poorly addressed by the healthcare system. We expect that this intervention will induce long-lasting positive effects. Moreover, this study will achieve its aim to elucidate the neurophysiological and biochemical mechanisms of this innovative intervention, and this knowledge will constitute a step forward in the development of effective telerehabilitation interventions. EARLY-COGN^3 could highlight potential predictors of treatment response, paving the way to more efficacious treatment pathways.

## Data Availability

No new data were created or analyzed in this study. Data sharing is not applicable to this article.
